# Herausforderungen der Automation bei der quantitativen Auswertung von Leberbiopsien: Automatische Quantifizierung von Leberverfettung

**DOI:** 10.1007/s00292-024-01298-6

**Published:** 2024-02-21

**Authors:** Jessica Darling, Nada Abedin, Paul K. Ziegler, Steffen Gretser, Barbara Walczak, Ana Paula Barreiros, Falko Schulze, Henning Reis, Peter J. Wild, Nadine Flinner

**Affiliations:** 1 Dr. Senckenbergisches Institut für Pathologie, Universitätsklinikum, Goethe-Universität Frankfurt, Theodor-Stern-Kai 7, 60596 Frankfurt am Main, Deutschland; 2 Medizinische Klinik 1, Universitätsklinikum, Goethe-Universität Frankfurt, Frankfurt am Main, Deutschland; 3 Frankfurt Cancer Institute (FCI), Frankfurt am Main, Deutschland; 4 German Organ Procurement Organization (DSO), Frankfurt am Main, Deutschland; 5 Frankfurt Institute for Advanced Studies (FIAS), Frankfurt am Main, Deutschland; 6 Wildlab, Universitätsklinikum Frankfurt MVZ GmbH, Frankfurt am Main, Deutschland; 7 University Cancer Center (UCT) Frankfurt-Marburg, Frankfurt am Main, Deutschland

**Keywords:** Computergestützte Bildinterpretation, Machine Learning, Nichtalkoholische Fettlebererkrankung, Betrachtervariation, Reproduzierbarkeit von Ergebnissen, Computer-assisted image interpretation, Machine learning, Non-alcoholic fatty liver disease, Observer variation, Reproducibility of results

## Abstract

**Hintergrund:**

Die MASLD (metabolische Dysfunktion-assoziierte steatotische Lebererkrankung, oder nichtalkoholische Fettlebererkrankung [NAFLD]) ist eine häufige Erkrankung, deren Diagnose auf der lichtmikroskopischen Auswertung von Leberbiopsien basiert. Diese unterliegt jedoch einer großen Interbetrachtervariabilität (IBV), die durch Hinzunahme von automatisierten Methoden verringert werden kann.

**Ziel der Arbeit:**

Ein Großteil der bestehenden computerbasierenden Methoden reflektiert nicht das, was in der Realität vom Pathologen bewertet wird. Ziel ist es, aufzuzeigen, wie diese Unterschiede die Vorhersage des Verfettungsgrads (VG) beeinflussen. Zusätzlich erschwert die IBV die Validierung von Algorithmen.

**Material und Methoden:**

Insgesamt 40 Gewebeschnitte wurden automatisch mit Bildanalysemethoden zur Fett‑, Zellkern- und Fibroseerkennung ausgewertet. Die Daten wurden verwendet, um den VG zu berechnen. Die IBV bei der Quantifizierung des VG wurde dabei an 18 Gewebeschnitten durch unterschiedliche Pathologen analysiert.

**Ergebnisse:**

Flächenbasierte Ansätze erzielten stärkere Korrelationen als zellkernbasierte Methoden (⌀ Spearman-Rho [ρ] = 0,92 vs. 0,79). Die Hinzunahme von Informationen zur Gewebekomposition verringerte für flächenbasierte und zellkernbasierte Methoden den durchschnittlichen absoluten Vorhersagefehler um 0,5 % bzw. 2,2 %. Unser finaler flächenbasierter Algorithmus, der Informationen zum Gewebeaufbau integriert, erreichte eine hohe Genauigkeit (80 %) und starke Korrelation (⌀ ρ = 0,94) mit der manuellen Auswertung.

**Diskussion:**

Die automatische und deterministische Bestimmung des VG lässt sich durch die Integration von Informationen der Gewebekomposition verbessern und kann dazu dienen, den Einfluss der IBV zu verringern.

**Zusatzmaterial online:**

Die Online-Version dieses Beitrags (10.1007/s00292-024-01298-6) enthält Abb. S1–S12.

## Abstract (en)

**Background:**

Metabolic dysfunction-associated steatotic liver disease (MASLD), or non-alcoholic fatty liver disease (NAFLD), is a common disease that is diagnosed through manual evaluation of liver biopsies, an assessment that is subject to high interobserver variability (IBV). IBV can be reduced using automated methods.

**Objectives:**

Many existing computer-based methods do not accurately reflect what pathologists evaluate in practice. The goal is to demonstrate how these differences impact the prediction of hepatic steatosis. Additionally, IBV complicates algorithm validation.

**Materials and methods:**

Forty tissue sections were analyzed to detect steatosis, nuclei, and fibrosis. Data generated from automated image processing were used to predict steatosis grades. To investigate IBV, 18 liver biopsies were evaluated by multiple observers.

**Results:**

Area-based approaches yielded more strongly correlated results than nucleus-based methods (⌀ Spearman rho [ρ] = 0.92 vs. 0.79). The inclusion of information regarding tissue composition reduced the average absolute error for both area- and nucleus-based predictions by 0.5% and 2.2%, respectively. Our final area-based algorithm, incorporating tissue structure information, achieved a high accuracy (80%) and strong correlation (⌀ Spearman ρ = 0.94) with manual evaluation.

**Conclusion:**

The automatic and deterministic evaluation of steatosis can be improved by integrating information about tissue composition and can serve to reduce the influence of IBV*.*

NAFLD (nichtalkoholische Fettlebererkrankung; neu: metabolische Dysfunktion-assoziierte steatotische Lebererkrankung, MASLD) ist eine hochprävalente Erkrankung, die durch manuelle lichtmikroskopische Auswertung von Leberbiopsien evaluiert wird. Diese Analyse unterliegt jedoch einer hohen Interbetrachtervariabilität (IBV), die durch automatische Bildverarbeitungsverfahren reduziert werden kann. Dabei müssen grundlegende Unterschiede zwischen automatischer und manueller Auswertung kritisch beleuchtet und ausgeglichen werden.

Die NAFLD betrifft weltweit etwa 25 % der Erwachsenen [16]. Sie umfasst die nichtalkoholische Fettleber (NAFL) und die nichtalkoholische Steatohepatitis (NASH). NAFL ist die nicht alkoholbedingte Verfettung von mehr als 5 % der Hepatozyten. NASH beinhaltet zusätzlich die Ballonierung der Hepatozyten und eine mögliche Fibrose des Gewebes. Der diagnostische Goldstandard ist die durch Pathologen ausgewertete Leberbiopsie [3]. Dazu wird häufig das vom NASH Clinical Research Network (NASH-CRN) entwickelte Bewertungssystem bestehend aus dem „NAFLD Activity Score“ (NAS) und dem Fibrosestadium verwendet [13]. Der NAS beschreibt Verfettungs-, lobuläre Entzündungs-, und hepatozelluläre Ballonierungsmerkmale. Die Grenzen der semiquantitativen NAS-Verfettungswerte (NAS-VW) werden festgelegt durch den Grad der Hepatozyten mit makrovesikulären Verfettung (0: < 5 %, 1: ≥ 5–33 %, 2: > 33–66 %, 3: > 66 %) [13]. Dennoch unterliegt die manuelle Auswertung durch das NASH-CRN-Bewertungssystem einer hohen IBV [4, 5, 12], die durch den Einsatz von Bildverarbeitungsmethoden reduziert werden kann. In dieser Arbeit werden Unterschiede zwischen automatischer und manueller Auswertung von Leberbiopsien erläutert und Methoden vorgestellt, die diese überbrücken können.

## Interbetrachtervariabilität

**Figure 1:**
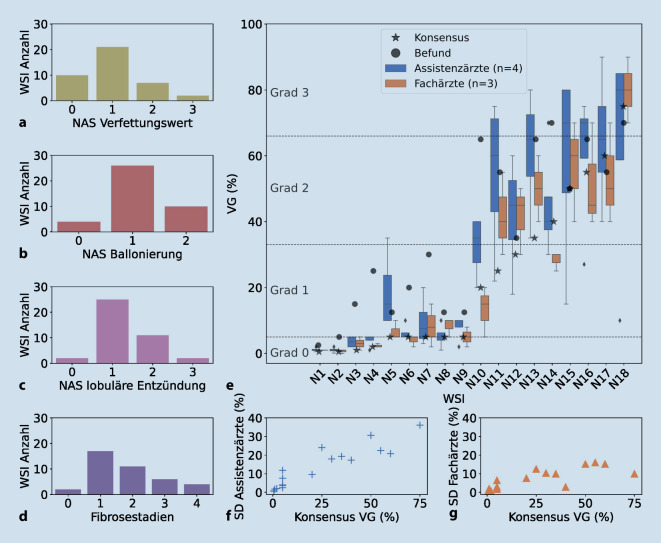

Interbetrachtervariabilität (IBV) bezeichnet das Phänomen, dass bei der Auswertung desselben Objekts verschiedene Betrachter voneinander abweichen können. Die Auswertung von NASH-Gewebeschnitten leidet unter diesem Problem [4, 5, 12]. Das wurde durch die Analyse der eigenen Kohorte des Dr. Senckenbergischen Instituts für Pathologie (SIP) erneut bestätigt (Abb. 1, Tab. 1).

**Table 1:** 

	Alle Betrachter (inkl. Konsensus)	Assistenzärzte, Befund, Fachärzte	Assistenzärzte	Fachärzte	Befund
Genauigkeit (%)	–	63,9 ± 9,6	56,9 ± 4,6	74,1 ± 6,9	61,1
Spannweite VG (%)	35,1 ± 21,8	35,1 ± 21,8	28,5 ± 23.3	12,9 ± 10,6	–
Durchschnittlicher (⌀) absoluter Fehler (%)	–	10,4 ± 3,3	11,8 ± 2,3	7,0 ± 1,4	14,8
ICC [95 %-CI], *p*	0.8 [0,6–0,9], 2,0e-45	0,8 [0,6–0,9], 6,0e-38	0,7 [0,5–0,9], 2,2e-13	0,9 [0,8–1], 3,0e-15	–

*CI* Konfidenzintervall,* ICC* Intraklassenkorrelationskoeffizient, *VG* Verfettungsgrad

Die SIP-Kohorte besteht aus Patienten, die mit Verdacht auf NASH im Zeitraum 2015–2020 biopsiert wurden. Die Leberbiopsien wurden mit einer 20fach- (0,275 µm/Pixel) oder 40fach-Vergrößerung (0,139 µm/Pixel) von einem Sysmex-Scanner (Sysmex Deutschland GmbH, Norderstedt, Deutschland) digitalisiert, um Whole Slide Images (WSI) zu erhalten. WSI der SIP-Kohorte wurden anhand der Befundung für die manuelle und automatische Reevaluation selektiert. Insgesamt wurden 20 WSI für die IBV-Untersuchung selektiert, wobei 2 WSI aus den 8 Kategorien (0–5 %, 6–15 %, …, ≥ 66 %) zufällig gezogen wurden. Zusätzlich wurden 4 Proben kategorienunabhängig gezogen. Von den 20 WSI hatten jedoch nur 18 WSI eine ausreichende Bildqualität für die Evaluation der Verfettung. Für die Konsensusevaluation wurden weitere 22 WSI hinzugefügt: Es wurden alle verbleibenden Bilder der SIP-Kohorte mit extremem Befund NAS-VW (NAS-VW 3, *n* = 2) verwendet und 20 weitere WSI kategorienunabhängig gezogen. Die meisten der insgesamt 40 Proben haben mittlere NAS-Werte für Verfettung, Ballonierung, Entzündung und Fibrose, aber auch die Extreme sind vorhanden und spiegeln somit die Verteilung der Verfettungsgrade von der gesamten SIP-Kohorte wider (Abb. 1a–d, Abb. S1). Diese 40 Fälle wurden von einem Expertengremium (F. Schulze, H. Reis, P. J. Wild) gemeinsam reevaluiert. Die Experten sichteten jedes WSI gleichzeitig auf einem Bildschirm. Jeder Pathologe evaluierte den VG, und Diskrepanzen wurden besprochen, um für jedes WSI einen gemeinsamen VG im Konsensusverfahren festzulegen. Die Ergebnisse dieser Auswertung werden als Goldstandard betrachtet und im Rest des Artikels als Konsensus bezeichnet. Für die Analyse der IBV wurden 18 der 40 WSI durch 7 Ärzte (3 Fach- und 4 Assistenzärzte für Pathologie) getrennt voneinander ohne gemeinsame Beratung ausgewertet.

Durchschnittlich erreichten die Ärzte eine Genauigkeit von 63,9 % für den NAS-VW und eine Spannweite von 35,1 % für den VG (Tab. 1). Mit einem Intraklassenkorrelationskoeffizient (ICC) von 0,7–0,9 ergab sich insgesamt eine gute Übereinstimmung innerhalb und zwischen den Bewertergruppen (Tab. 1), die etwas höher ist als in anderen Studien mit ICCs von teils nur 0,55 [5]. Weiterhin zeigte sich vor allem mit zunehmendem VG ein größerer Wertebereich und größere Standardabweichung (Abb. 1e–g), was mit vorherigen Untersuchungen übereinstimmt [5]. Dies ist besonders problematisch, da sich die Werte teils sogar über 3 NAS-VW erstreckten (N11, N14 und N15; Abb. 1e). Assistenzärzte hatten im Durchschnitt eine signifikant höhere VG geschätzt als Fachärzte und Konsensus (Wilcoxon-Test für verbundene Stichproben, *p* = 0,004 bzw. 0,0003). Auch dies stimmt mit vorangegangenen Studienergebnissen überein, in welchen ein weniger erfahrener Pathologe signifikant höhere VG schätzte als ein erfahrenerer Pathologe (Kappa = 0,62) [12]. Auch die Teststärke kann durch die IBV verändert werden, wenn nicht alle Patienten die Einschlusskriterien wirklich erfüllen [4]. Automatisierte Methoden können verwendet werden, um den Einfluss der IBV zu reduzieren und solche Vorfälle zu verhindern.

## Bestehende Automatisierungsmethoden zur Erkennung der Verfettung

Verschiedene Studien beschäftigen sich mit der Quantifizierung von VG in WSI, wobei viele dieser Methoden flächenbasierte Messungen verwenden [5, 7, 10, 11]. Die Grundprinzipien dieser Methoden können in drei Schritten zusammengefasst werden:

1. Erkennung von Gewebeflächen und Identifikation von weißen Objekten im Gewebe (z. B. Fettvakuole oder Lumen der Gefäße),
2. Klassifizierung dieser Objekte als „Fett“ oder „Nicht-Fett“ basierend auf ihre morphologischen Merkmale durch Setzen von Schwellenwerten [5, 7] oder Strategien des Maschinellen Lernens [11, 15],
3. Bestimmung des Verhältnisses von Gewebefläche zur Summe der Flächen von Fettvakuolen.

**Figure 2:**
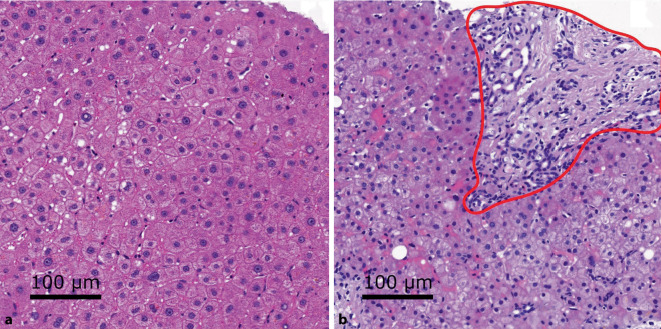

Der entscheidende Unterschied zwischen manueller und automatischer flächenbasierter Auswertung ist, dass Pathologen den VG auf den Anteil der verfetteten Hepatozyten bzw. die auf die von Hepatozyten bedeckte Fläche beziehen [13] und dass die rein flächenbasierte Betrachtung des Gesamtgewebes vor allem bei einer erkrankten Leber problematisch ist, da der Krankheitsprozess zu Veränderungen im Gewebe führt [2]. Die gesunde Leber besteht vorwiegend aus Hepatozyten, wobei eine erkrankte Leber große fibrotische, nichthepatozytäre Flächen vorweisen kann (Abb. 2). Diese Areale müssten also bei flächenbasierten Methoden exkludiert werden, um korrekte Ergebnisse zu erhalten. Deshalb wurde bereits die Verteilung der VG in kleinen Kacheln von Homeyer et al. analysiert, allerdings werden hierbei nichtverfettete und nichthepatische Regionen gleichbehandelt [11]. Ein weiterer Ansatz ist die zellkernbasierte Bildverarbeitung, bei der die relative Anzahl an Fettvakuolen (mit bestimmten Flächengrößen Grenzen) zur Anzahl der Zellkerne normalisiert wurde, um den VG zu bestimmen [15].

Allerdings ist unklar, ob automatische flächen- oder zellkernbasierte Methoden besser geeignet sind, um den VG der Pathologen zu bestimmen, wenn Informationen über die Gewebekomposition miteinbezogen werden. Im Folgenden wurden Gewebeschnitte automatisiert analysiert und die verschiedenen Ansätze miteinander verglichen.

## Automatisierungsprozess zur Gewebeanalyse

**Figure 3:**
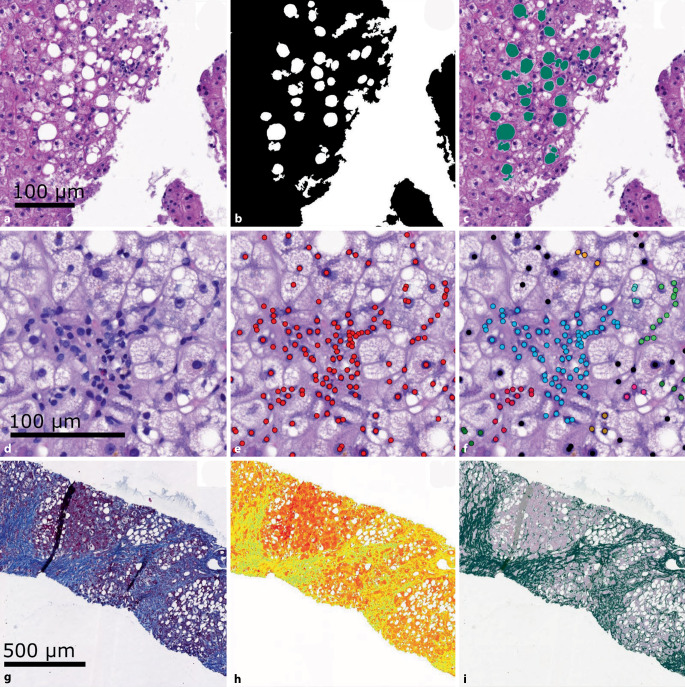

Unser Automatisierungsprozess kann in 3 Schritte aufgeteilt werden: die Fett- und Zellanalysen und die Quantifizierung von Kollagen (Abb. 3, Abb. S2–S4). Für die Fett- und Zellanalysen wurden Hämatoxylin-Eosin(HE)-gefärbte Gewebeschnitte verwendet, für die Kollagenquantifizierung Masson-Trichrom-gefärbte Serienschnitte. Diese Gewebeschnitte wurden dann automatisch analysiert, um das kollagenproportionale Areal (CPA) zu bestimmen [1]. Die Trennung von Vorder- und Hintergrundpixel in der Fetterkennung und CPA-Bestimmung erfolgte jeweils durch die Berechnung eines globalen Otsu-Schwellenwertes anhand der Pixelinformation. Hierzu wurde das WSI in einer reduzierten Auflösung (4,5 µm/Pixel) verwendet.

### Fetterkennung

Nach der Berechnung des globalen Otsu-Schwellenwerts anhand der Varianz der Farbinformation der Pixel wurde das WSI in Kacheln unterteilt (~ 1500 × 1500 µm^2^, entspricht 5456 Pixel in 20fach-Vergrößerung bzw. 10.781 Pixel in 40fach-Vergrößerung. Verarbeitungsparameter werden automatisch der Pixelanzahl angeglichen) und in der höchsten Auflösungsstufe analysiert (Abb. 3a). Der Schwellenwert wurde auf jede Kachel angewendet und trennte somit Vorder- und Hintergrund (Abb. 3b). Weiße Objekte wurden anhand ihrer morphologischen Merkmale durch vorgegebene Schwellenwerte (Abb. S2) als „Fett“ oder „Nicht-Fett“ klassifiziert (Abb. 3c). Dabei wurden nur makrovesikuläre Fettvakuolen (≥ 40 µm^2^) berücksichtigt. Pro Kachel wurden die Gewebe- und Fettfläche sowie die Anzahl der Fettvakuolen festgehalten.

### Zellkernanalyse

In jeder Kachel wurden außerdem die Zellkerne mittels HoVer-Net, einem Convolutional Neuronal Network, unter Verwendung der Kumar-Gewichte detektiert ([9, 14]; Abb. 3d, e). Gesundes Leberparenchym besteht zum Großteil aus regelmäßig verteilten Hepatozyten [2]. Eine untypisch dichtere Verteilung deutet darauf hin, dass die Zellen in diesem Gebiet keine Hepatozyten sind und nicht in eine zellkernbasierte Analyse einbezogen werden sollten. Diese dichten Regionen stellen meist entzündete Areale oder andere Strukturen mit größerer Zellkerndichte (z. B. Gefäße oder Gallengänge) dar. Um diese Zellkerngruppen zu erkennen, wurden die Positionen der erkannten Zellkerne mittels des KD-Baums (k = 15 µm), einer Methode zur Bestimmung der nächsten Nachbarn, analysiert. Gruppen mit mehr als 3 Zellkernen wurden als nichthepatozytär definiert (Abb. 3f). Theoretisch bietet HoVer-Net unter Verwendung weiterer Gewichtungen auch eine direkte Klassifizierung der Zellkerne. Dies führt jedoch zu Übersegmentierung (z. B. Erkennung granulozytärer, zytoplasmatischer Anteile als Zellkerne) und falscher Klassifizierung (z. B. wurden gesunde Hepatozyten als neoplastische Zellen erkannt), und wurde deshalb nicht verwendet (Abb. S5; [8, 9]).

### CPA-Bestimmung

Nach der Berechnung des globalen Otsu-Schwellenwerts anhand der Sättigung der Pixel wurde das WSI in Kacheln aufgeteilt und in höchster Auflösung bearbeitet (Abb. 3g). Gewebeflächen wurden durch Anwendung des Schwellenwerts erkannt (Abb. 3h). Im nächsten Schritt wurden Gewebepixel anhand Ihrer Farbe in 2 Gruppen mittels k‑Means-Clustering unterteilt (Abb. 3i). Kollagen wird in der Masson-Trichromfärbung bläulich und Parenchym rötlich dargestellt. Hierbei werden auch physiologische, kollagenhaltige Strukturen der Leber (z. B. Kapsel, Gefäße, Portalgebiete) mit angefärbt. Die „blaue“ Pixelgruppe wurde summiert und zum Gesamtgewebe, inklusive Fettflächen, in Relation gesetzt, um das CPA zu berechnen.

## Bestimmung des Verfettungsgrads

**Figure 4:**
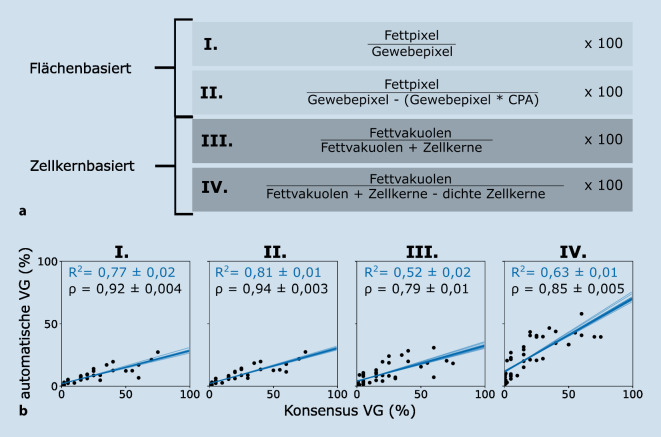

Um zu klären, ob eine flächen- oder zellkernbasierte Methode den manuell bestimmten VG von Pathologen am besten widerspiegelt, wurden verschiedene Verhältnisse basierend auf den Daten aus der Bildverarbeitung gebildet (Abb. 4a). Methode I ist die rein fettflächenbasierte Messung der VG, Methode II berücksichtigt zusätzlich noch fibrotische Areale. Methode III ist zellkernbasiert, berücksichtigt jedoch keine Zelltypen. Methode IV zieht von der Gesamtzellzahl alle Zellen in dichten Arealen ab, um z. B. Entzündungszellen nicht mitzuberücksichtigen. Bei den Methoden III und IV wird die Anzahl detektierter Fettvakuolen zur Gesamtzellzahl addiert, da die Zellkerne verfetteter Hepatozyten oft nicht in der Schnittebene liegen.

Auffällig an den über Methode I bis III generierten VGs war, dass diese im Vergleich zur menschlichen Auswertung zu niedrig ausfielen. Dieser systematische Bias in flächenbasierten Methoden, der in mehreren Studien [5, 7, 10] beobachtet wurde, lässt sich z. B. damit erklären, dass rein flächenbasierten Methoden niemals den durch die Skala vorgegebenen Maximalwert (100 %) erreichen können, was zu einer artifiziellen Verzerrung der Skala führt [10]. Methode IV hingegen erreichte als einzige den manuellen Wertebereich, hatte dabei aber eine schwächere Korrelation als flächenbasierte Methoden (Abb. 4b). Die flächenbasierte Methode II erreichte hingegen die höchste Korrelation zum Konsensus (R^2^ = 0,81, ρ = 0,94) und zeigte keine Unterschiede für 20fach und 40fach vergrößerte WSIs (Abb. S6, S7). Somit ist Methode II besser geeignet als zellkernbasierte Methoden, um den Pathologen-VG widerzuspiegeln (Abb. 4b). Methode II erreichte zusätzlich eine stärkere Korrelation als andere flächenbasierte Methoden (ρ = 0,82 bzw. 0,92) [5, 10]. Auch Nativ et al. haben gezeigt, dass höhere R^2^-Werte durch flächen- im Vergleich zu zellkernbasierten Methoden erreicht werden, wobei allerdings noch keine Gewebekomposition berücksichtigt wurde [15]. Die Hinzunahme von Informationen zum Gewebeaufbau verbesserte die Bestimmung des VGs sowohl für flächen- (ρ = 0,92 vs. 0,94) als auch für zellkernbasierte (ρ = 0,79 vs. 0,85) Methoden (Abb. 4b).

Gewebekompositionsbeschreibende Informationen wurden zusätzlich auf Zusammenhänge mit dem aus der Befundung entnommenen Fibrosestadium und NAS lobulärer Entzündungswert untersucht. Allgemein gilt, dass höhere Fibrosestadien durch hohe CPA-Werte widergespiegelt wurden (Abb. S8A). Allerdings ist das Fibrosestadium im Gegensatz zum CPA keine quantitative Messung, sondern eine Beschreibung der Architektur der fibrotischen Veränderung im Gewebe [13]. Ein Zusammenhang zwischen NAS lobulären Entzündungswerten und dem Anteil von zellkerndichten Arealen wurde ebenfalls beobachtet (Abb. S8B). Die zellkerndichten Areale sind jedoch ebenfalls keine direkte Vorhersage des NAS lobulären Entzündungswerts, da dieser die Anzahl an entzündlichen Foki in einem 200fach-Sichtfeld darstellt [13]. Weiterhin ist es nicht möglich, zwischen dicht gelegenen Entzündungszellen und anderen Zellen mit dichter Zellkernverteilung (z. B. Gefäß- und Gallengangepithel) sowie zwischen portaler und lobulärer Lokalisation zu differenzieren. Allerdings war das Ziel der Zellkernverteilungsanalyse nicht ausschließlich, Entzündungszellen zu identifizieren, sondern hepatozytäre und nichthepatozytäre Zellen zu unterscheiden, um damit den NAS-VW automatisch bestimmen zu können.

**Figure 5:**
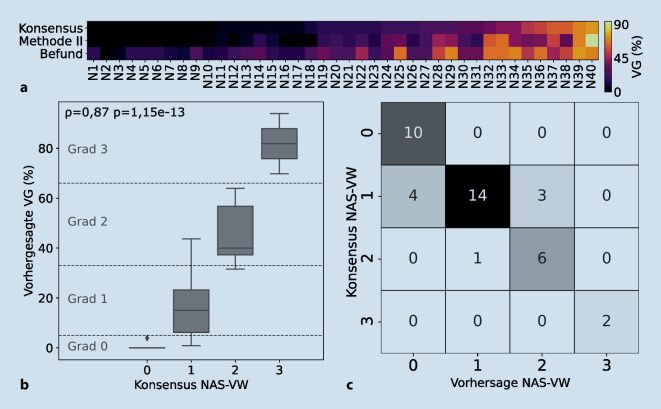

**Figure 6:**
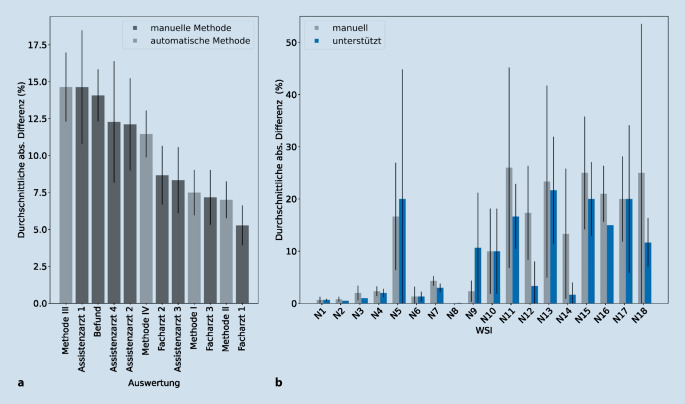

Um automatisierte und manuelle Auswertungen vergleichbar zu machen, wurde eine lineare Regression durchgeführt, da die automatisch bestimmten Werte trotz hoher Korrelation aufgrund ihres niedrigeren Wertebereichs nicht direkt mit den manuell bestimmten Werten verglichen werden können. Die durch Methode II bestimmten Werte bilden also nicht direkt den Pathologen-VG ab, können allerdings nachträglich in diesen umgerechnet werden (Abb. S9). Basierend auf den umgerechneten Werten wurde nun der absolute Fehler (Differenz zwischen vorhergesagtem und Konsensus-VG) und die Genauigkeit (Vergleich vorhergesagter NAS-VW und Konsensus-NAS-VW) berechnet. Die Genauigkeit der Methode II auf den 18 WSIs der IBV-Umfrage lag mit 66,7 % höher als die durchschnittliche Genauigkeit der Assistenzärzte, Befunde und Fachärzte (63,9 %; Tab. 1). Darüber hinaus übertraf die Genauigkeit von Methode II auf der gesamten Kohorte (40 WSI) mit 80 % (⌀ absoluter Fehler 8,4 %; Tab. 2) zusätzlich die Genauigkeit des Befunds mit 57,5 % (⌀ absoluter Fehler 14,1 %, Abb. 5a). Die Korrelation zwischen vorhergesagten VG und Konsensus-NAS-VW war statistisch signifikant (ρ = 0,87, *p* = 1,15e-13) und stärker als in einer im Jahr 2020 publizierten Studie (ρ = 0,66, *p* < 0,001) [7]. Die Mehrheit der Vorhersagen lagen innerhalb der entsprechenden NAS-VW-Grenzen (Abb. 5b) und der vorhergesagte NAS-VW weichte um maximal einen Wert ab (Abb. 5c). Beim Vergleich der durchschnittlichen absoluten Fehler aller zur Verfügung stehenden Daten hatte Methode II eine niedrigere Abweichung vom Konsensus als 6 Pathologen und die Befundung (Abb. 6a). Die flächenbasierte Methode II mit Integration von Gewebekompositionsinformationen stellt somit eine akkurate und vollständig automatisierte Methode zur Quantifizierung der Verfettung dar.

**Table 2:** 

Methode	Genauigkeit (%)	Durchschnittlicher absoluter Fehler (%)
I	80,0	8,4 ± 9,3
II	80,0	7,9 ± 7,3
III	57,5	17,3 ± 13,1
IV	62,5	15,1 ± 8,1

Um zu untersuchen, ob die durch Methode II bestimmten VGs zur Verringerung der IBV beitragen können, wurden die 18 WSIs aus der IBV-Evaluation durch 3 Assistenzärzte erneut bewertet, wobei ihnen diesmal der automatisch berechnete VG zur Orientierung angezeigt wurde. Insgesamt hat der Einsatz von Methode II zu einer Verbesserung geführt: Die durchschnittliche absolute Abweichung zum Konsensus ist um 2,9 % gesunken. Für 8 Fälle kam es zu keiner Änderung (± 1 %), für 8 Fälle kam es zu einer Verbesserung der bestimmten VGs, während es nur bei 2 Fällen zu einer Verschlechterung kam (Abb. 6b). Vor allem für Fälle mit hohen VGs hilft der automatisch bestimmte Wert. Hier wurden Verbesserungen um bis zu 13,9 % (N12) beobachtet. Dies zeigt, dass durch die Unterstützung von computergestützten Methoden genauere und reproduzierbarere Ergebnisse in der manuellen Auswertung von Gewebeschnitten erzielt werden konnten.

## Schlusswort

Grundsätzlich erreichen in unserer Arbeit die automatisierten flächenbasierten Methoden bessere Ergebnisse als zellkernbasierte Methoden bei der Bestimmung von makrovesikulärer Verfettung. Durch Integration von Informationen zur Gewebekomposition lässt sich die Zuverlässigkeit der VG-Vorhersagen zusätzlich verbessern (Tab. 2). Somit ließen sich durch Methode II nicht nur reproduzierbare, sondern sogar genauere VG als in den ursprünglichen Befunden berechnen (Abb. 6a).

Um den gegenwärtigen NAS-VW korrekt und automatisiert abzubilden, muss also eine robuste und akkurate Fettsegmentierung sowie die Erkennung von nichthepatozytär besiedelten Flächen gewährleistet werden. Die hier vorgestellte Methode zur Identifizierung dieser Flächen durch dichte Areale ist allerdings eine Approximation und eine direkte Klassifizierung von Zellen könnte genauere Ergebnisse liefern. Da vorhandene Klassifizierungs- und Segmentierungsmethoden jedoch nicht zuverlässig funktionieren (Abb. S5), sind neue Trainingsdaten notwendig, deren Herstellung sehr zeitaufwendig sein kann. Weiterhin ist es wünschenswert, wenn Informationen zur Fibrose sowie auch Informationen zur Entzündung gleichzeitig in flächenbasierte Methoden integriert werden könnten, da beide essenzielle Bestandteile des Gewebes darstellen. Dieses kann erreicht werden, wenn die Fibrose ebenfalls im HE-Schnitt detektiert wird, um zu verhindern, dass die berechnete CPA im Parallelschnitt durch z. B. abgeschwommenes Gewebe verfälscht wird oder sich CPA und andere nichthepatozytäre Regionen überschneiden (und somit doppelt gewertet werden).

Die automatisch bestimmten Werte können Pathologen bei der manuellen Analyse zur Orientierung dienen und die IBV minimieren (Abb. 6b), was die Qualität der Patientenversorgung langfristig steigern kann. Allerdings wurde bisher nicht gezeigt, ob rein digitale Biomarker (ganz ohne IBV) oder digital assistierte Analysen besser dazu geeignet sind, das Krankheitsstadium zu beschreiben. Eine solche Studie könnte auch die entsprechende Eignung flächenbasierter und zellbasierter Auswertungen zur Beschreibung des Krankheitsverlaufs vergleichen. Außerdem ist zu beachten, dass der Umrechnungsprozess von automatisch bestimmten VG auf eine menschlich nachvollziehbare Skala selbst auch auf manuellen, möglicherweise IBV-beeinflussten Auswertungen basiert. Ein standardisierter digitaler Biomarker könnte diesen Umrechnungsprozess verzichtbar machen.

Die Quantifizierung von Fett spielt auch in anderen Gebieten eine Rolle, wie z. B. bei Lebertransplantationen, bei denen Organe mit einer makrovesikulären Verfettung von über 30 % nur eingeschränkt geeignet sind [6]. Auch hier wäre es interessant zu untersuchen, inwiefern automatisierte Methoden die Patientenversorgung verbessern könnten und ob die hier vorgestellten Methoden, die für NAFLD-Schnitte entwickelt wurden, übertragbar sind. Außerdem könnten solche Methoden in Zukunft auch bei der Ausbildung von Pathologen verwendet werden, um zuverlässigere und akkuratere Vorhersagen zu ermöglichen. Eine steigende Qualität manueller Auswertungen ist zur Entwicklung moderner und genauerer Algorithmen hilfreich, welche dann auch regulär im klinischen Alltag angewendet werden können.

## Fazit für die Praxis

- Die manuelle Auswertung von Gewebeschnitten für Verfettung ist anfällig für Interbetrachtervariabilität (IBV).
- Wie unsere Studie demonstriert, hat die automatisierte Bestimmung des Verfettungsgrads (VG) das Potenzial, genauere Ergebnisse als eine typische manuelle Analyse zu liefern. Hierzu sind folgende Punkte zu beachten. 1. Gute Kommunikation zwischen Pathologen und Algorithmenentwicklern ist essenziell, da z. B. das Einbeziehen von Informationen zur Gewebekomposition die Qualität der Auswertung steigert. 2. Flächenbasierte Methoden reproduzieren den Pathologen-NAFLD-Activity-Score-Verfettungswert(NAS-VW) akkurater als zellkernbasierte Methoden, erfordern jedoch eine Anpassung des Wertebereichs.
- Die Hinzunahme von automatisch bestimmten Werten zur Orientierung während der Befundung verringert die IBV und nähert die manuell bestimmten Werte an den Konsensus an.
- Weitere Untersuchungen könnten zeigen, ob zellkern- oder flächenbasierte Methoden zur Beantwortung anderer klinischen Fragestellungen besser geeignet sind.

### Supplementary Information




