# Supplementary material for: Challenges of automation in quantitative evaluation of liver biopsies: Automatic quantification of liver steatosis
Source: Pathologie (Heidelb). 2024 Feb 21;45(2):115–23. [Article in German] doi: 10.1007/s00292-024-01298-6 (PMC10901975; doi:10.1007/s00292-024-01298-6)
Supplement: Supplementary file 1 [file 292_2024_1298_MOESM1_ESM.pdf]

Onlinezusatzmaterial:

Abb. S1 - S12

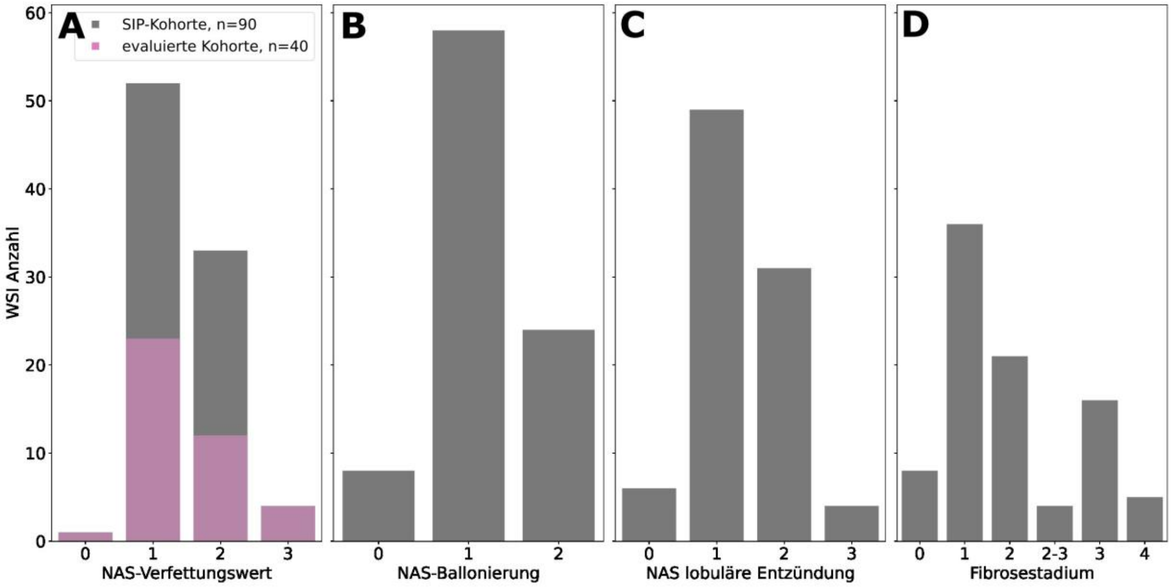

Abb. S1: Charakterisierung der Dr. Senckenbergischen Institute für Pathologie (SIP)-Kohorte. Die in **A-D** gezeigten Informationen wurden von der Befundung entnommen. In A wird der Anteil der evaluierten WSI in Lila hervorgehoben.

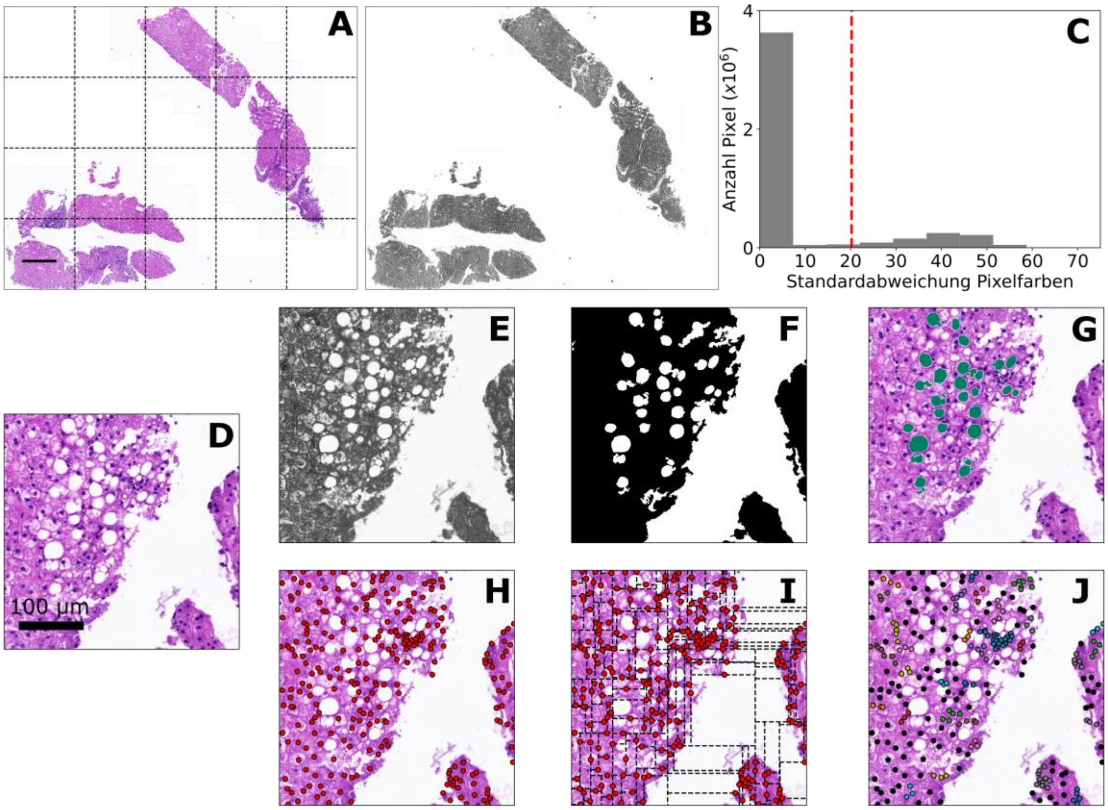

Abb. S2: Übersicht der automatisierten Bildanalyse von H&E WSI. A Das gesamte „whole slide image“ (WSI) wird als RGBA-Bild geöffnet in einer Auflösung von ~4,5 µm^2/Pixel. Anschließend wird das gesamte WSI, erneut, in der höchsten Auflösung, geöffnet und in Kacheln geteilt (siehe Schritt D). Die Maßstableiste zeigt 500 µm an. **B** Für jeden Pixel des WSI in der kleineren Auflösung wird die Standardabweichung der Farbkanäle bestimmt. **C** Basierend auf dem Histogramm der Standardabweichungen aller WSI-Pixel wird der Otsu Schwellenwert bestimmt. **D** Aus dem WSI werden Kacheln in der Größe (~ 1500 x 1500 µm^2) in der höchsten Auflösung extrahiert. Der hier gezeigte Ausschnitt hat eine Größe von 420 x 420 µm^2. **E** Die Standardabweichungen der Pixelfarben wird berechnet um den global bestimmter Schwellenwert des Standardabweichens verwenden zu können. Dadurch werden Pixel in Vorder- und Hintergrund getrennt. **F** Das Bild wird in mehreren Schritten „gereinigt“. Kleine weiße Objekte (≤ 40 µm^2), die sich im Vordergrund (i.e. im Gewebe) befinden werden entfernt, und ein binäres Schließen wird durchgeführt. Ein Hysteresis Algorithmus wird angewendet, um die wahren Grenzen der Fettobjekte, die durch die Binarisierung oft verkleinert werden, wiederherzustellen. Ein Watershed Algorithmus wird verwendet, um verbunden weiße Objekte zu trennen. **G** Erkannte weiße Objekte werden durch ihre morphologischen Merkmale z.B. Fläche (≥40µm^2 und ≤10000µm^2), die Ausdehnung (Verhältnis Objektpixel zu Pixel des Begrenzungsrahmen) (≥ 0,5), und Achsenverhältnis (≤ 2) in „Fett“ oder „nicht-Fett“ klassifiziert. **H** Zellkerne werden durch das HoVer-Net detektiert [2]. Dazu werden die Kumar Gewichte verwendet [3]. **I** Die Position der erkannten Zellkerne wird verwendet, um einen KD-Baum zu bilden. Durch diesen Baum kann für jeden Zellkern benachbarte Zellkernen innerhalb einer bestimmten Distanz (15µm) bestimmt werden. **J** Diese Nachbarschaftsinformationen kann zur Analyse der verbundenen Komponentengruppen von dichten Zellkernen benutzt werden. Gruppen mit ≥ 3 Zellkernen werden als dichte Region klassifiziert.

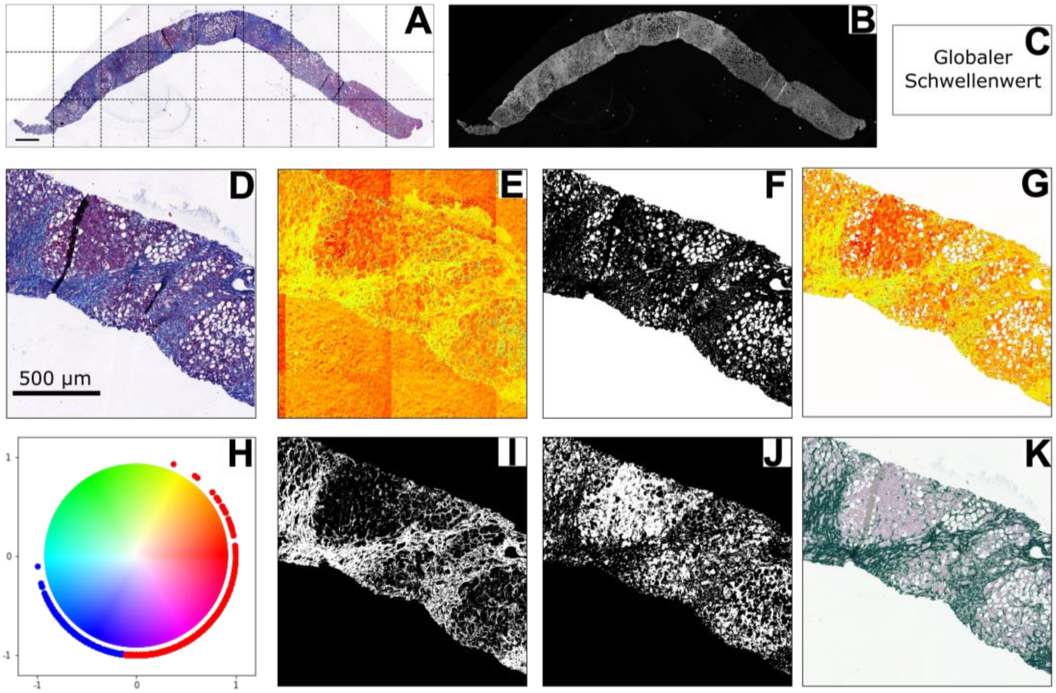

Abb. S3: Übersicht Kollagenproportionale Areal (CPA) Erkennung von Massen-Trichom WSI. A Das gesamte WSI wird als RGBA-Bild in einer Auflösung von ~4,5 µm/Pixel geöffnet. Anschließend wird das gesamte WSI in der höchsten Auflösung in Kacheln geteilt (siehe Schritt D). Die in **A** dargestellte Maßstableiste zeigt 1000 µm an. **B** Das WSI wird in den HSV-Farbraum konvertiert, um Sättigungsinformation der Pixel (hier abgebildet) zu gewinnen. **C** Diese Sättigungsinformation der Pixel wird verwendet, um einen globalen Otsu Schwellenwert zu bestimmen. **D** Die höchste Auflösung der WSI wird in Kacheln der Größe ~1.500 x 1.500 μm^2 geteilt. Der hier gezeigte Ausschnitt hat eine Größe von 210x210$\mu m.^2$ **E** Die Kachel wird in den HSV-Farbraum konvertiert. **F** Der global bestimmter Schwellenwert wird auf Kachelebene angewendet. Dazu wird die Sättigungsinformation der Kachelpixel verwendet. Dadurch wird ein binäres Bild, bestehend aus Vorder- und Hintergrund produziert. Kleine Objekte im Hintergrund (kleine Gewebsstücke, Artefakte etc.) werden entfernt. Wenn mindestens 1% der Bildfläche aus Vordergrundpixel (Gewebe) besteht, wird die Kachel weiterverarbeitet. Hierdurch werden relevante Gewebeflächen als Vordergrundpixel identifiziert. **G** Die Farbtoninformationen der im Schritt F erkannten Gewebeflächen wird als Grundlage für den K-means Algorithmus verwendet. **H** Im HSV-Farbraum wird Farbinformation als Grad (°) repräsentiert. Das bedeutet z.B., dass rote Farbtöne sich jeweils bei ~ 0 ° und 360 ° befinden. Farbinformationen werden in den kartesischen Raum konvertiert, sodass ähnliche Farben beieinander liegen. Dann werden Pixel mittels dem K-means Algorithmus in k=2 Gruppen aufgeteilt. Als Initialisierungspunkte wird jeweils ein Punkt im blauen und im roten Farbraum verwendet. Aufgrund der hohen Anzahl der Pixel wurde ein Downsampling für die Visualisierung durchgeführt. **I, J** Es wird pro Pixelgruppe eine binäre Maske generiert. Pixel der Gruppe 1 sind in **I** abgebildet, und Pixel der Gruppe 2 in **J. K** Um zu identifizieren, welche Gruppe die blauen Pixel repräsentiert, wird für jede Pixel in der Gruppe die Distanz zu dem initialen „blauen" Punkt berechnet. Die Gruppe mit der kleinsten durchschnittlichen Distanz wird durch blaue Pixel identifiziert. Der Anteil des Kollagens wird berechnet, in dem das Verhältnis zwischen blauen Pixeln durch die Gesamtpixel (inklusiv Fettvakuolen, erkannt im analogen Prozess wie in Supp. Abb. 1 dargestellt, mit der Ausnahme von der Bestimmung des Schwellenwerts, welcher durch die Sättigungsinformation berechnet wird) gebildet wird.

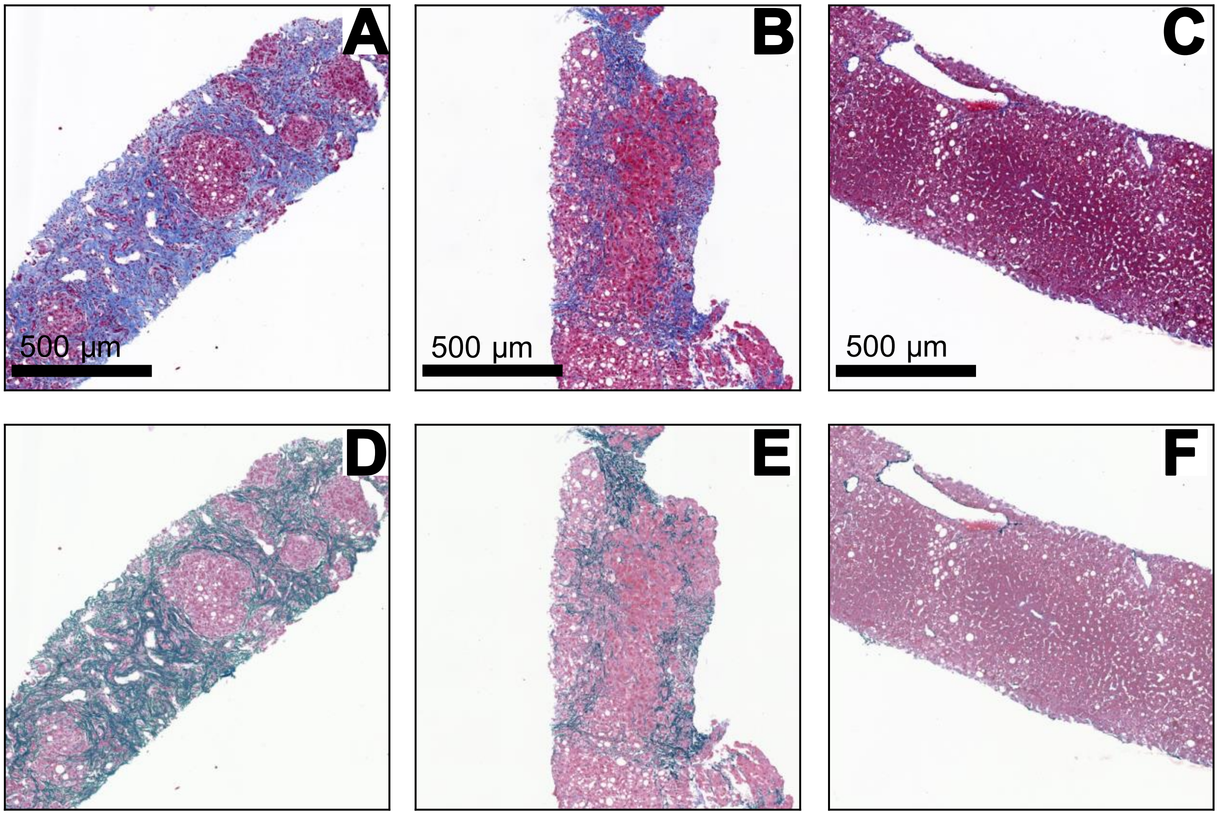

Abb. S4: Beispiel der CPA-Berechnung. **A, B** und **C** zeigen Ausschnitte von Masson-Trichrom gefärbten WSI. **D, E** und **F** heben Pixel, die kollagenhaltige Strukturen abbilden, in Grün hervor. **A** und **D** zeigen einen Ausschnitt eines stark fibrotisiertes WSI, **B** und **E** dagegen ein etwas weniger fibrotisiertes WSI. **C** und **F** stellen ein Ausschnitt eines WSI dar, der keine Fibrose, sondern nur kleine Stellen mit strukturellen Kollagen, aufweist.

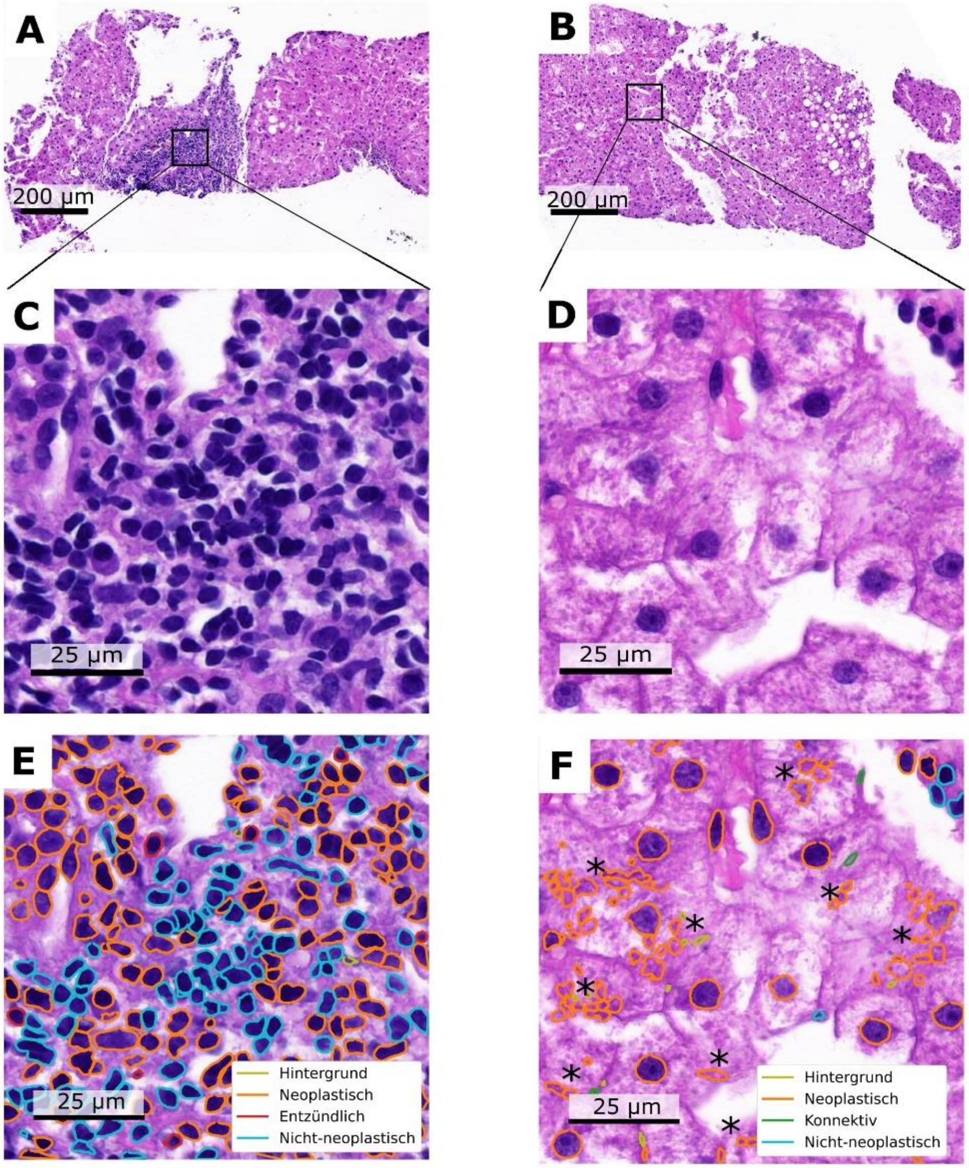

Abb. S5: Fehlerhafte Zellkernklassifizierung und Segmentierung. Das HoVer-Net bietet, z.B. unter Verwendung der PanNuke Gewichtungen, die Möglichkeit erkannte Zellkernen nach Zelltyp zu klassifizieren [1, 2]. Allerdings gab es bei der Anwendung dieser Funktion zahlreiche fehlerhafte Klassifizierungen und Übersegmentierungen. **A** und **B** zeigen Ausschnitte eines H&E gefärbte WSI. **C** und **D** stellen jeweils Vergrößerungen von Ausschnitten **A** und **B** dar, die in **E** und **F** unter Verwendung der PanNuke Gewichtungen segmentiert und klassifiziert wurden [1, 2]. Der vorhergesagte Zellkerntyp wird durch die farbliche Umrandung der Zellkerne angezeigt. Auffällig sind insbesondere fehlerhaften Zellklassifizierungen in E, sowie auch die zahlreichen granulozytäre, zytoplasmatische Anteile, die fälschlich als Zellkerne identifiziert wurden. Diese übersegmentierten Gebiete sind in F mit einem Stern (*) markiert. Aus diesem Grund wurde für zellkernbasierten Methoden (Methoden III und IV) die Zellkernsegmentierung durch Anwendung der Kumar Gewichtungen durchgeführt, die allerdings keine Zellkernklassifikation ermöglicht.

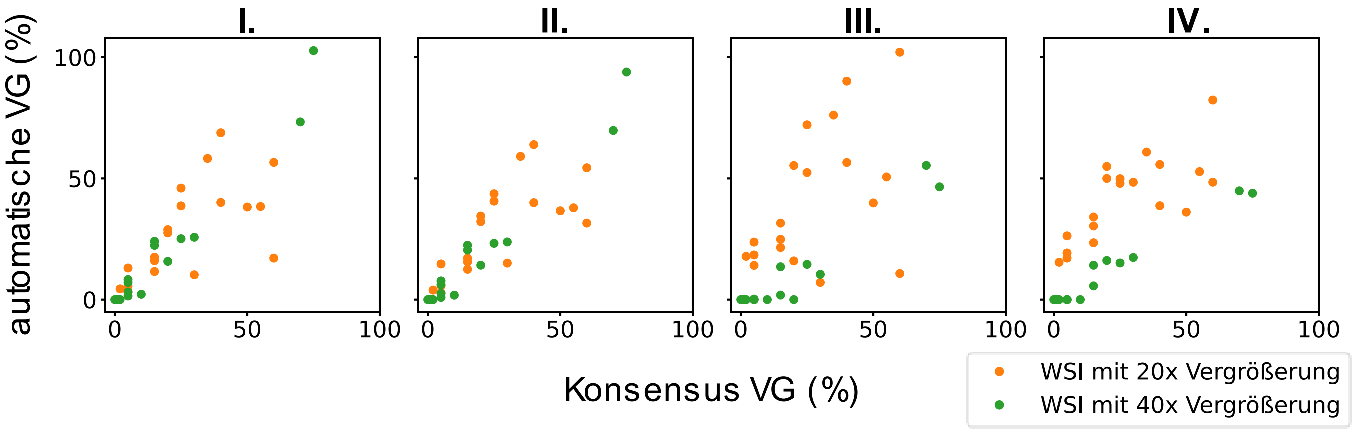

Abb. S6: Einfluss der Vergrößerung. Abgebildet ist das Verhältnis zwischen automatisch bestimmten VG und Konsensus VG für Methoden I bis IV. Die Vergrößerung der WSI wird durch die Farbe des Punktes angegeben.

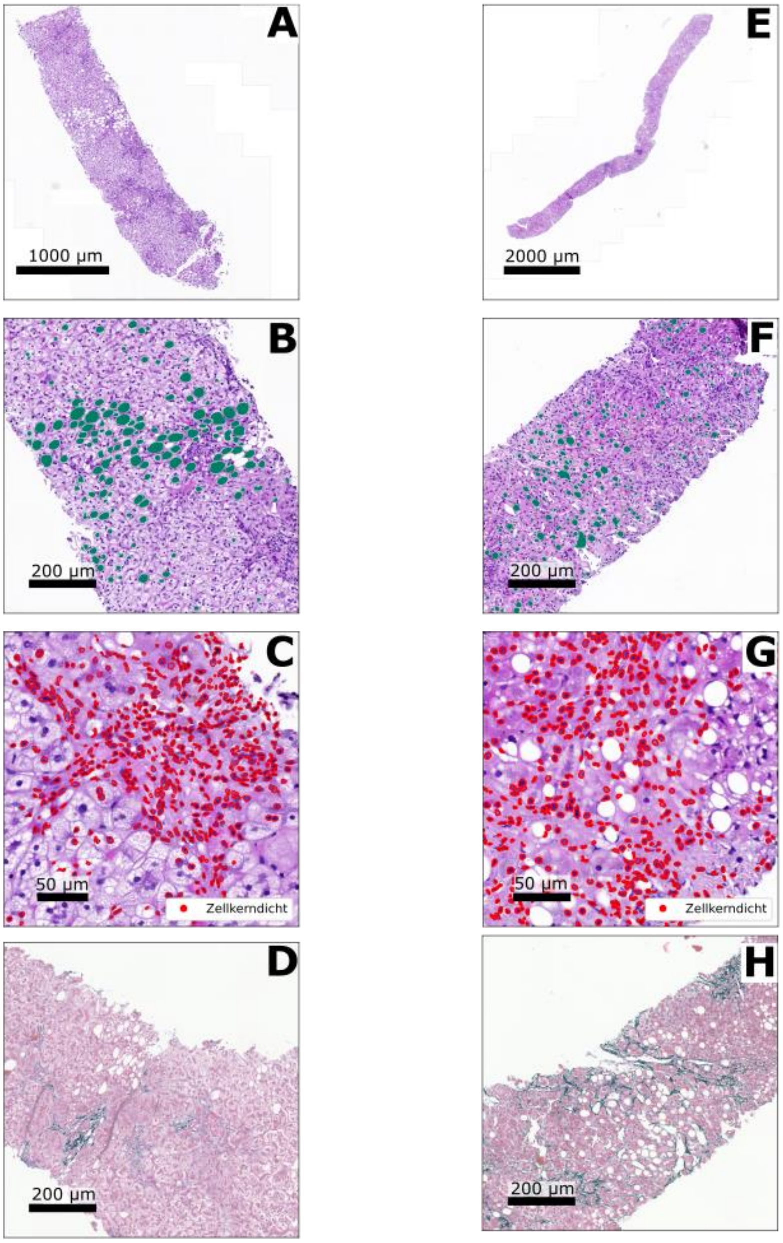

Abb. S7: Vergleich der Verarbeitung mit unterschiedlichen Vergrößerungen. Hier werden Schritte der Bildverarbeitung für 20x (links) und 40x (rechts) vergrößerte WSI exemplarisch dargestellt. Dabei zeigen **A** & **E** die unbearbeiteten WSI-Ausschnitte, **B** & **F** die Fetterkennung (erkannte Fettvakuolen sind Grün markiert), **C** & **G** die Zellkernerkennung, und **D** & **H** die CPA-Bestimmung (erkannte Fibrose in Grün hervorgehoben)

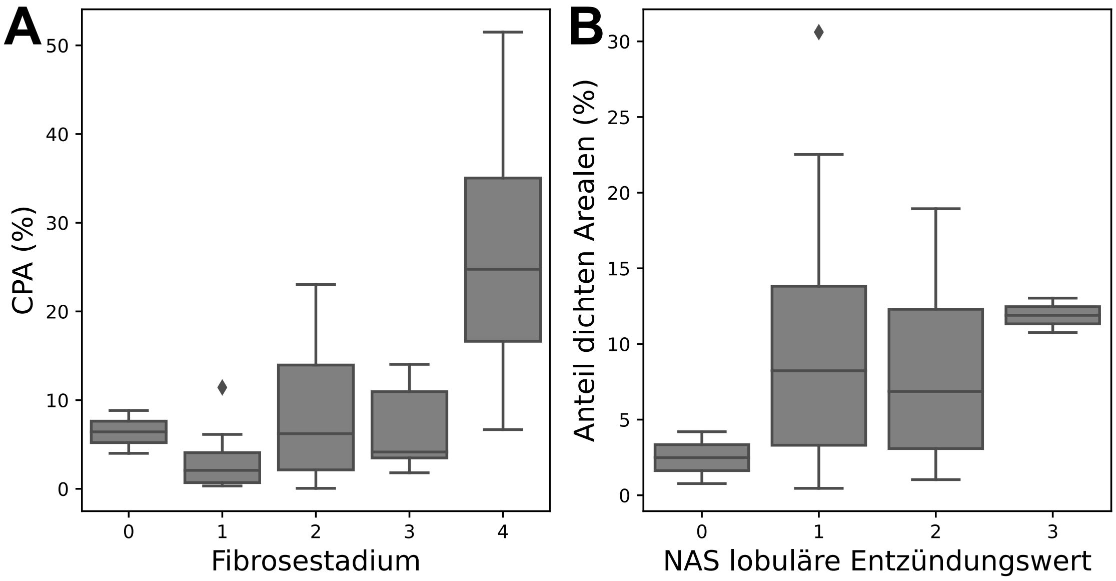

Abb. S8: Beziehung zu weiteren histologischen Merkmalen. Dabei werden Fibrosestadium und NAS lobuläre Entzündungswerte aus der Originalbefundungen entnommen. **A** zeigt das Verhältnis zwischen Fibrose Stadium (wobei Stadien 1a, b, und c zu Stadium 1 zusammengefasst werden) und CPA (Spearman Rho = 0,44, p = 0,005). **B** zeigt das Verhältnis zwischen dem Anteil der Gewebefläche, dass durch dichtbesiedelte Zellkerne gebildet wird, und dem NAS lobulärer Entzündungswert (Spearman Rho = 0,29, p = 0,065). Um den Anteil dichter Arealen zu bestimmen wird ein konvexes Vieleck um Zellkerne in dicht besiegelte Gruppen mit ≥ 3 Zellkernen gebildet. Die Gewebeflächen (ohne Fettflächen) die sich innerhalb dieser Vielecke befinden wird summiert und in Verhältnis zur Gesamtfläche gesetzt.

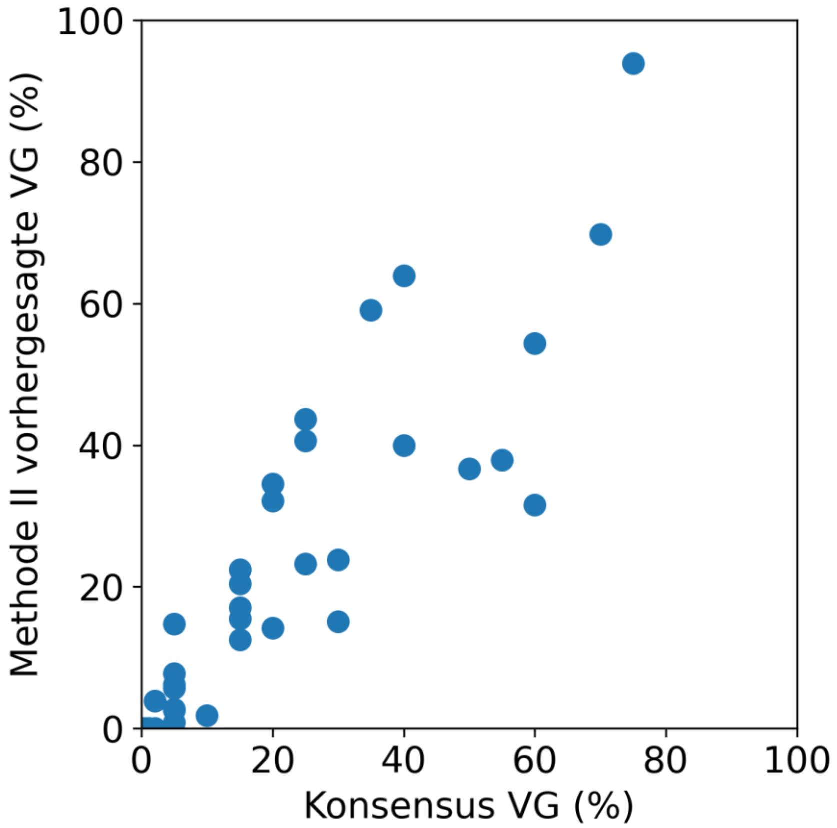

Abb. S9: Vorhergesagte VG der Methode II. Die vorhergesagten VG stellen dabei die durch die lineare Regression umgerechnete Werte dar.

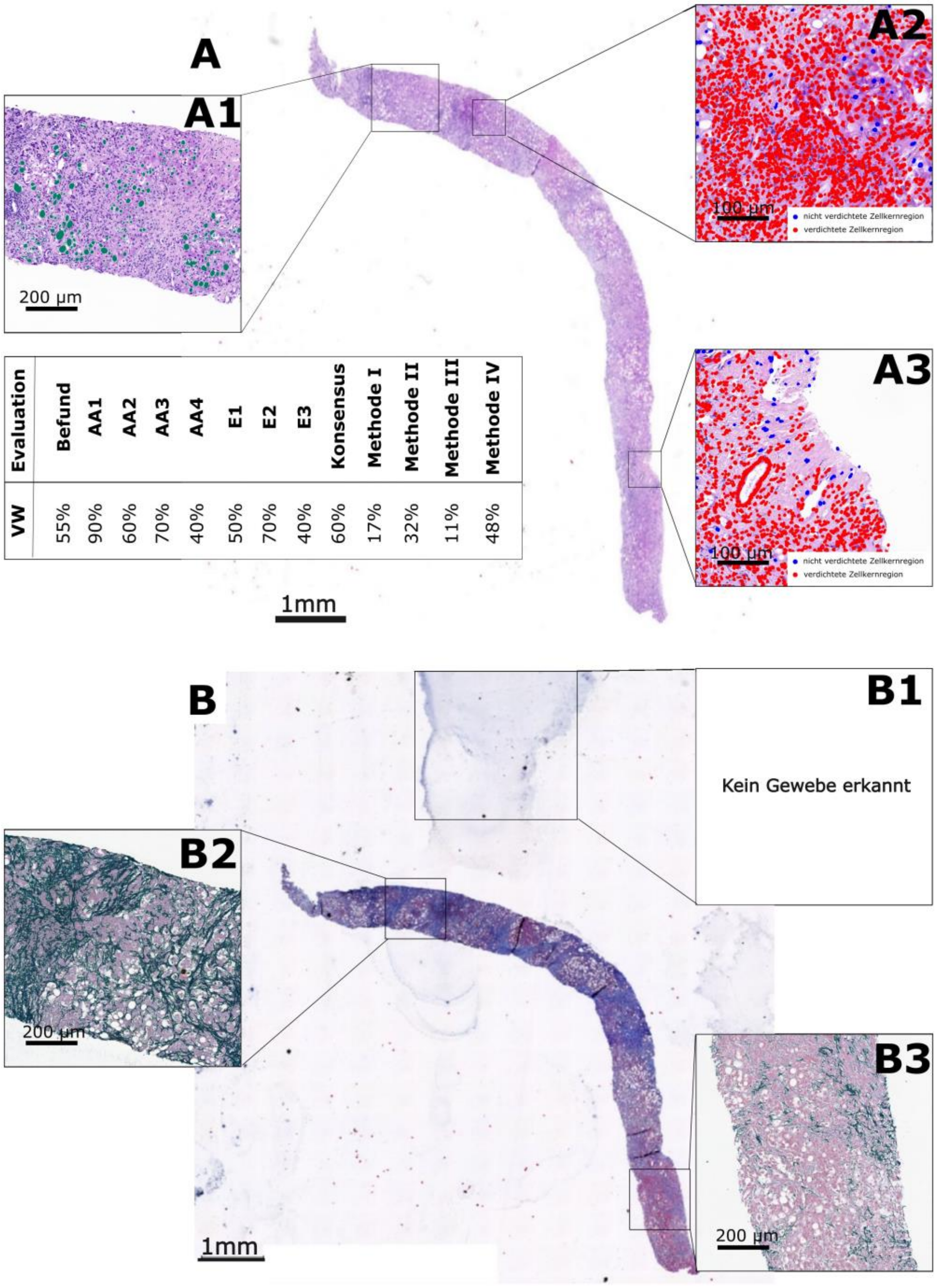

Abb. S10: WSI mit der größten absoluten Differenz (%) zum Konsensus VG. Um eine übersichtliche Visualisierung zu gewährleisten, werden hier Ausschnitte des WSIs gezeigt. **A** zeigt das H&E gefärbte WSI, **B** das Masson-Trichrom gefärbte WSI. **A1** zeigt erkannte Fettvakuolen in Grün an. **A2** und **A3** zeigen die Zellkernanalyse. Blaue Konturen zeigen Zellkerne in Hepatozyten-ähnliche Verteilung, währen rote Konturen Zellkerne in dichten Arealen markieren. **B1, B2** und **B3** zeigen die CPA an, wobei bei **B1** keine Analyse durchgeführt wurde, weil keine ausreichende Gewebefläche erkannt wurde. Fibrotische Areale werden in Grün hervorgehoben.

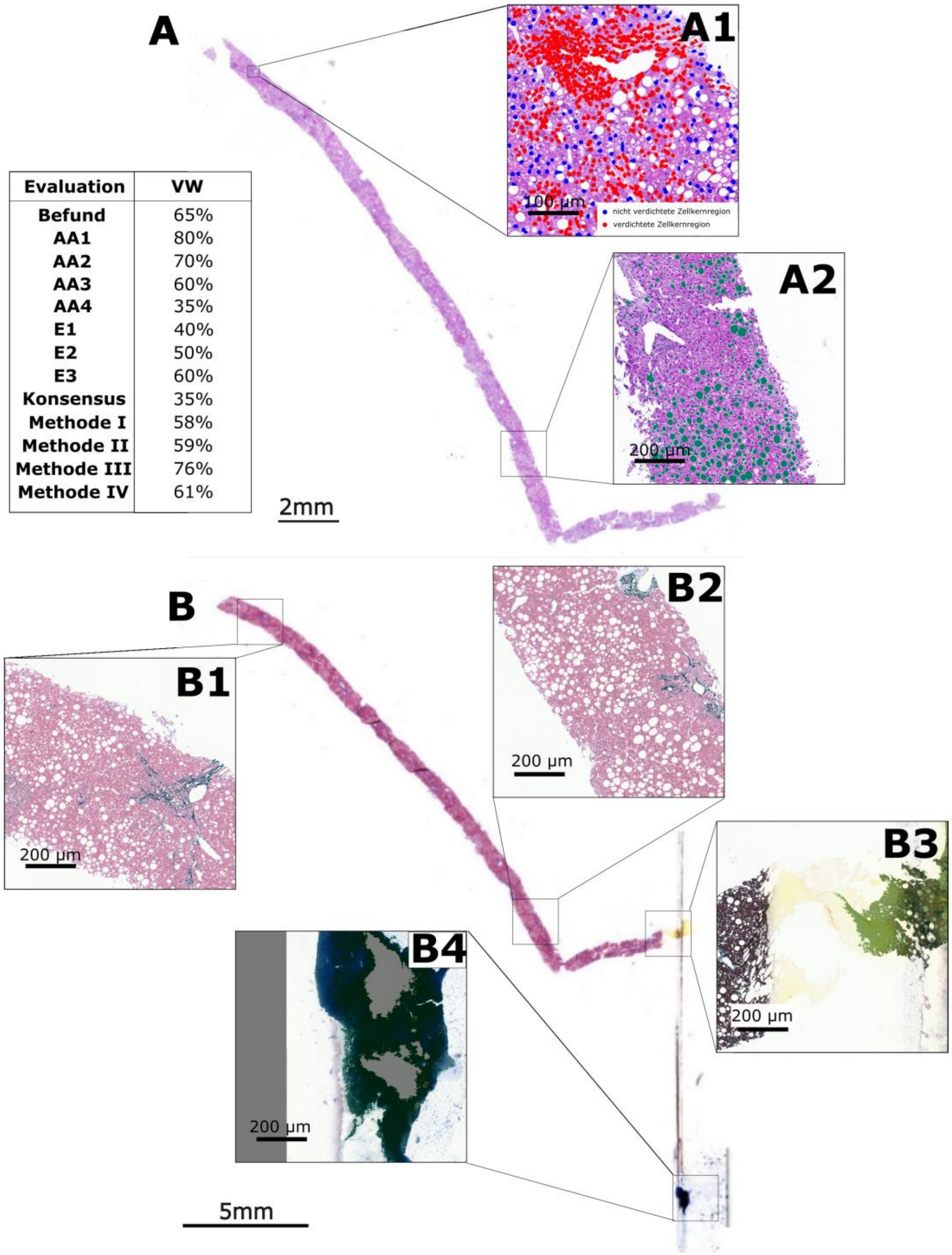

Evaluation	VW
Befund	65%
AA1	80%
AA2	70%
AA3	60%
AA4	35%
E1	40%
E2	50%
E3	60%
Konsensus	35%
Methode I	58%
Methode II	59%
Methode III	76%
Methode IV	61%

Abb. S11: WSI mit der zweitgrößten absoluten Differenz (%) zum Konsensus VG. Um eine übersichtliche Visualisierung zu gewährleisten werden hier Ausschnitte des WSIs gezeigt. **A** zeigt das H&E gefärbte WSI, **B** das Masson-Trichrom gefärbte WSI. **A1** zeigt die Zellkernanalyse. Blaue Konturen zeigen Zellkernen in Hepatozyten-ähnliche Verteilung, währen rote Konturen Zellkerne in dichten Arealen markieren. **A2** zeigt erkannte Fettvakuolen in Grün an. **B1-B4** zeigen die CPA an, wobei fibrotische Areale in Grün hervorgehoben werden. **B3** und **B4** zeigen Areale mit Färbungsartifakte, wobei große Flächen fälschlich als Fibrose erkannt wurden. In **B4** werden nicht-eingescannte Areale in Grau gezeigt.

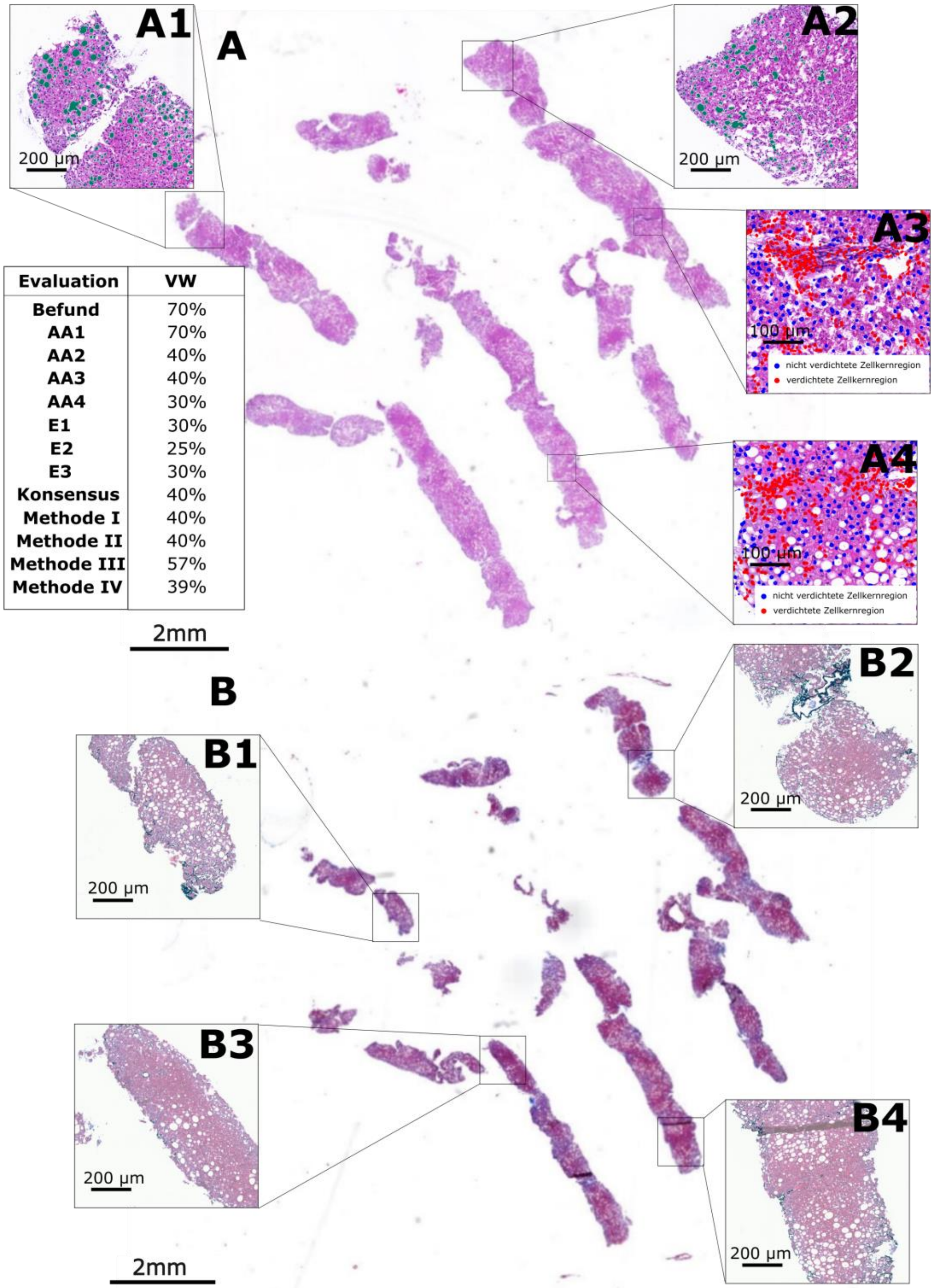

Evaluation	VW
Befund	70%
AA1	70%
AA2	40%
AA3	40%
AA4	30%
E1	30%
E2	25%
E3	30%
Konsensus	40%
Methode I	40%
Methode II	40%
Methode III	57%
Methode IV	39%

Abb. S12: WSI mit der kleinsten absoluten Differenz (%) zu Konsensus VG. Um eine übersichtliche Visualisierung zu gewährleisten werden hier Ausschnitte des WSIs gezeigt. **A** zeigt das H&E gefärbte WSI, **B** das Masson-Trichrom gefärbte WSI. **A1** und **A2** zeigen erkannte Fettvakuolen in Grün an. **A3** und **A4** zeigen die Zellkernanalyse. Blaue Konturen zeigen Zellkernen in Hepatozyten-ähnliche Verteilung, währen rote Konturen Zellkerne in dichten Arealen markieren. **B1-B4** zeigen die CPA an, wobei fibrotische Areale in Grün hervorgehoben werden.

Weitere Information

Der Quellcode wird unter diesem Link zur Verfügung gestellt: https://github.com/Je55ie555/steatosis-quantification

Literatur

1. Gamper J, Koohbanani NA, Benes K et al. (2020) PanNuke Dataset Extension, Insights and Baselines.
2. Graham S, Vu QD, Raza SEA et al. (2019) Hover-Net: Simultaneous segmentation and classification of nuclei in multi-tissue histology images. Medical Image Analysis 58:101563
3. Kumar N, Verma R, Sharma S et al. (2017) A Dataset and a Technique for Generalized Nuclear Segmentation for Computational Pathology. IEEE Trans. Med. Imaging 36:1550-1560
